# Antivirals for allosteric inhibition of Zika virus using a homology model and experimentally determined structure of envelope protein

**DOI:** 10.1186/s13104-017-2685-7

**Published:** 2017-07-28

**Authors:** Sandun Fernando, Teshan Fernando

**Affiliations:** Biological and Agricultural Engineering Department, TAMU, 303C, Scoates Hall, 2117, College Station, TX 77843 USA

## Abstract

**Objective:**

An approach to inhibiting enveloped flaviviruses is to deter the ability of the envelope protein(s) binding onto glycoproteins. In previous work, using a small ~100-amino acid homology model of Zika virus envelope protein (ZVEP), we proved the susceptibility of Zika virus to inhibition. In this work, we verify the efficacy of the homology model based antiviral search method using a larger protein (>400 amino acids) and comparing the results with the experimentally determined one (PDB ID:5IRE).

**Results:**

By examining how glycan molecules, small-molecule probes and screened ligands that have a high affinity to ZVEP, we report the mechanics of ZVEP to inhibition via allosteric blockage of the glycan-binding domain while proposing even more possibly potent inhibitors. The small molecular probes based study using the homology model and subsequently verified using actual experimental structure, 5IRE, revealed that ZVEP is druggable. A pharmacophore analysis followed by screening showed at least four ligands that allosterically binds to the glycan binding domain constituted by residues VAL 153 and ASN 154 in 5IRE. Based on further selection criteria ZINC40621658 was identified to have high potential to be a strong antiviral candidate for Zika virus inhibition.

## Introduction

The World Health Organization estimates that Zika disease, an infection caused by Zika virus, to reach epidemiological levels [1, 2]. An infection with the virus causes Zika Fever [3, 4], and yet, no vaccines or drugs are available to prevent or treat an infection [5]. The connections of the virus to microcephaly [1, 6] and neurological conditions in infected adults, including cases of the Guillain–Barré syndrome [7, 8], make Zika potentially destructive.

Among several approaches to developing antiviral drugs targeting the Zika flavivirus, inhibition of the envelop protein is considered to be promising. Computational and experimental work have proven that blocking envelope protein is a viable approach to inhibiting flaviviruses [9–11], which include Zika, Dengue, Encephalitis, and West Nile.

To perform in silico inhibitor screening, experimentally determined structures of the protein(s) responsible for virus’ virulence are essential; and in many instances, these structures are not available [12]. So, there is a need for a technique to reliably screen for drugs in the absence of three-dimensional structural data. Nevertheless, advances in biotechnology have enabled rapid availability of nucleotide sequence data that could be used to develop homology models that in turn could be utilized for inhibitor screening. This work looks at finding antivirals targeting ZVEP using a homology model and then comparing the efficacy of the potential inhibitors with the experimentally determined NMR structure of ZVEP 5IRE [13]. The methods can be applied to druggable proteins of any emerging pathogen even in the absence of complete structural information.

## Main text

**Fig. 1:**
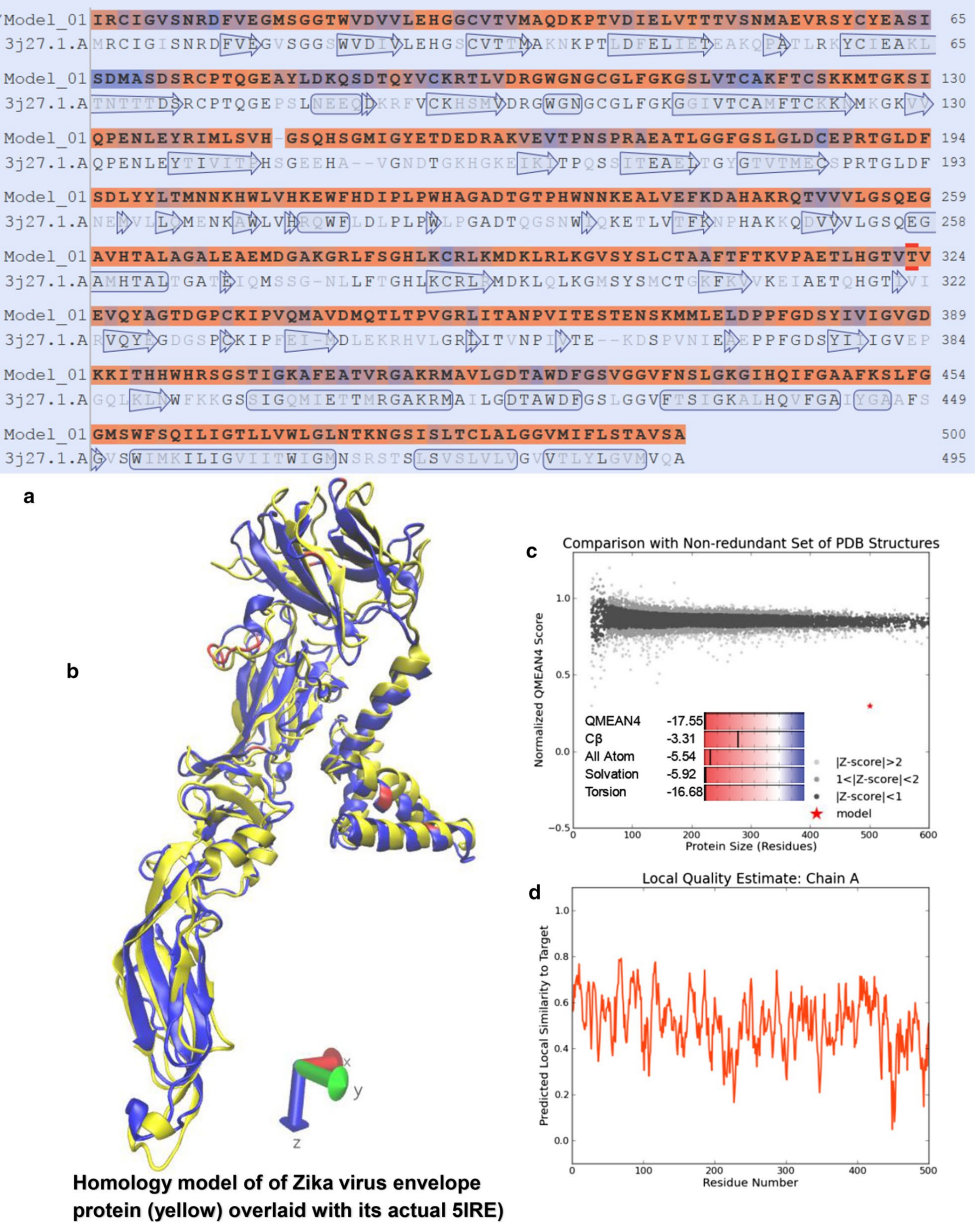
**a** A 115-amino-acid section of WNEP (3J27) aligned with ZVEP which was the best fit for developing the homology model after considering profile-function relationships; **b** homology model of 460—amino-acid section of Zika virus envelope protein homology model (*yellow*) overlaid with its actual crystal structure (*blue*). The model was created before the actual structure was released. **c**, **d** Various quality estimates of the predicted homology model

The amino acid sequence of ZVEP was resolved initially using Zika virus’ 10,794-nucleotide National Center for Biotechnology Information (NCBI) NC_012532.1 [14] via Basic Local Alignment Search Tool (BLAST) [15]. Then using the BLAST-protein module, sequences that matched the ZVEP were retrieved. This search was aided by a profile/function search performed using Interpro [16] embedded in Swiss-model [17] protein modeling tool. The result was a 500-amino-acid ZVEP segment that contained key domains likely responsible for glycosidic interactions (Fig. 1a). Then using SWISS-MODEL workspace [17], a template search with was performed using BLAST and HHBlits against the SWISS-MODEL Template Library (SMTL). The models with the highest quality based on the features of the target-template alignment were selected for model building.

Based on the target-template alignment, homology models were built via Promod-II [18]. Conserved coordinates between the target and the template were copied from the template to the model. Insertions and deletions were remodeled using a fragment library. Then, side-chains were rebuilt. Lastly, the geometry of the model was regularized by using force fields. The overall and per-residue model quality was assessed using the QMEAN4 [19] and Global Model Quality Estimation (GMQE) scoring functions as shown in Fig. 1c, d. Each residue is assigned a QMEAN reliability score between zero and one, describing the expected similarity to the native structure and higher numbers indicate a greater reliability of the residues. GMQE combines properties from the target-template alignment, and the score is expressed as above reflecting the expected accuracy of a model built with that alignment and template.

Dengue virus envelope protein (WNEP, PDB ID: 3j27) was identified to be the best template to be used for the development of the final ZVEP homology model and a three-dimensional model for the target protein was generated (Fig. 1b). Model quality assessment tools were used to assess the reliability of the resulting models are given in the inset (Fig. 1c, d). The predicted ZVEP homology structure (yellow—Fig. 1b), when overlaid with that of 5IRE for comparison (blue—Fig. 1b), shows >50% structural identity preserved indicating the model’s utility as a proxy for studying ZVEP behavior.

### Active site analysis

**Fig. 2:**
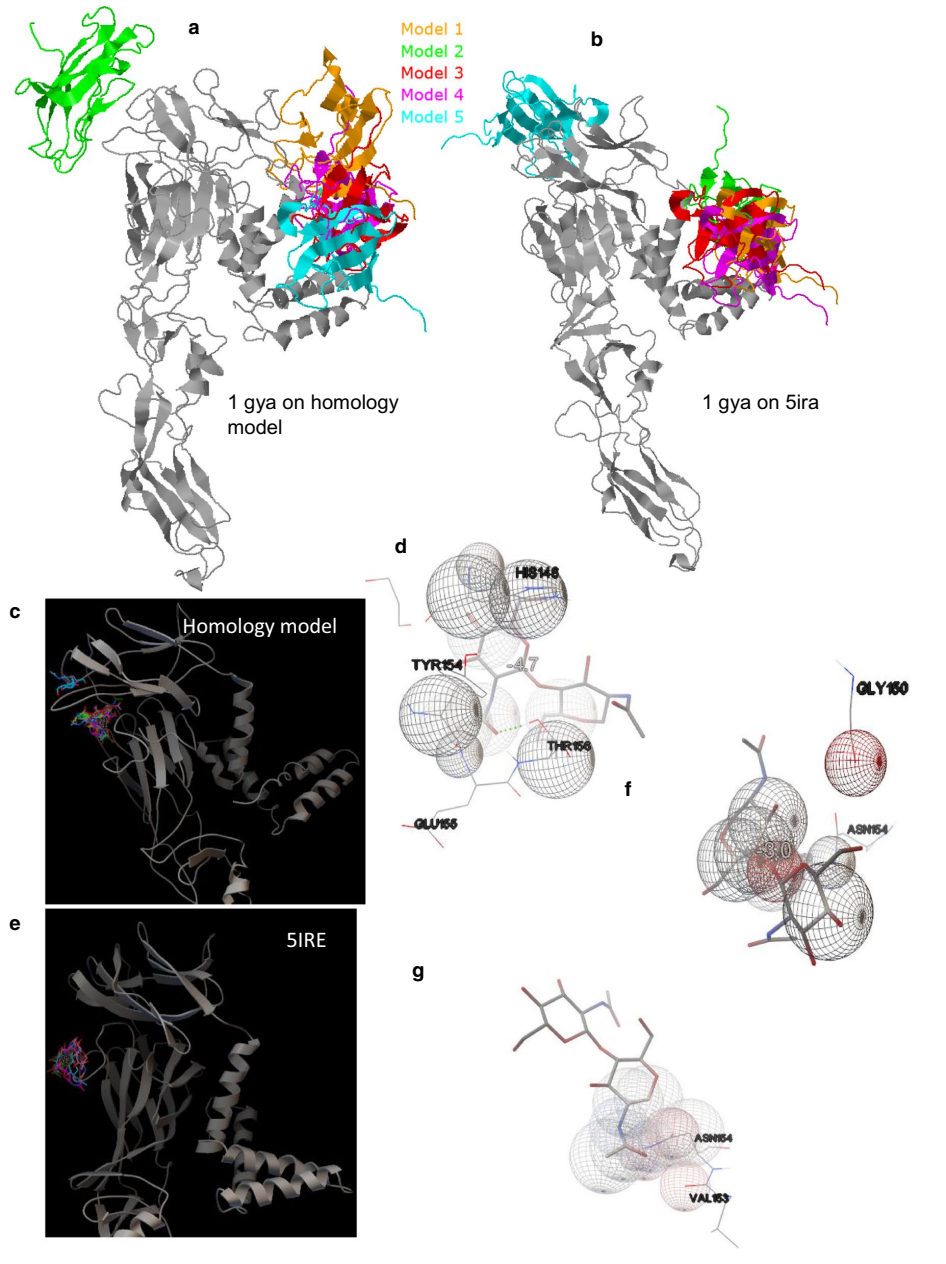
NAG-containing binding protein interacting with **a** ZVEP homology model and **b** 5IRE crystal structure; NAG binding confirmation distribution on **c** ZVEP homology model and **d** its ligand-receptor interactions; NAG binding confirmation distribution on **e** 5IRE and **f** its ligand receptor interactions; **g** PDB published ligand-receptor interactions NAG-5IRE. The numbers embedded in interaction diagrams are binding energies in kcal/mol

The next step was to identify the active site in the absence of any experimental structural data. For this initially, how an N-linked glycan fragment in the adhesion domain of human T lymphocyte glycoprotein CD2 (PDB ID: 1GYA) binds onto the ZVEP homology model was analyzed via Zdock. N-acetylglucosamine (NAG) is widely used as a model glycan molecule [20]. It was evident that the homology model predicted two 1GYA binding domains, one lying toward the exterior of the envelope while the more potent glycan binding domain (with multiple conformations) lying toward the interior of the envelope (Fig. 2a). A verification done with the real ZVEP 5IRE confirmed the existence of these two domains (Fig. 2b). Identification of the binding pockets of the homology model was made iteratively using protein–protein interaction data NAG binding site information from other flavivirus (e.g. West Nile 2I69, tick-bourne encephalitis 1SVB, and dengue 4UTC) envelope proteins via Autodock Vina while focusing on specific residues that were known to interact with NAG. Initially, the search area of the grid-box was made broader to isolate high-affinity biding domains; then, based on results, the search grids were gradually narrowed. Autodock Vina predicted the binding site to be between residues 154–156 (note that the specific amino-acids may be different in the homology model of the actual structure due to model variability) (Fig. 2c, d). A verification done using the same algorithm with ZVEP 5IRE (Fig. 2e) predicted the binding restudies to be ASN 154 (Fig. 2f). The experimental structure depicts the binding interactions with residues VAL 153 and ASN 154 (Fig. 2g). It should be noted that the models were able to locate binding sites with much higher affinity across the protein chain; however, these may be inaccessible (to glycan) due to the orientation of the chain in the envelope and spatial distribution of other proteins.

### Druggability assessment of homology model and experimental structure of ZVEP

**Fig. 3:**
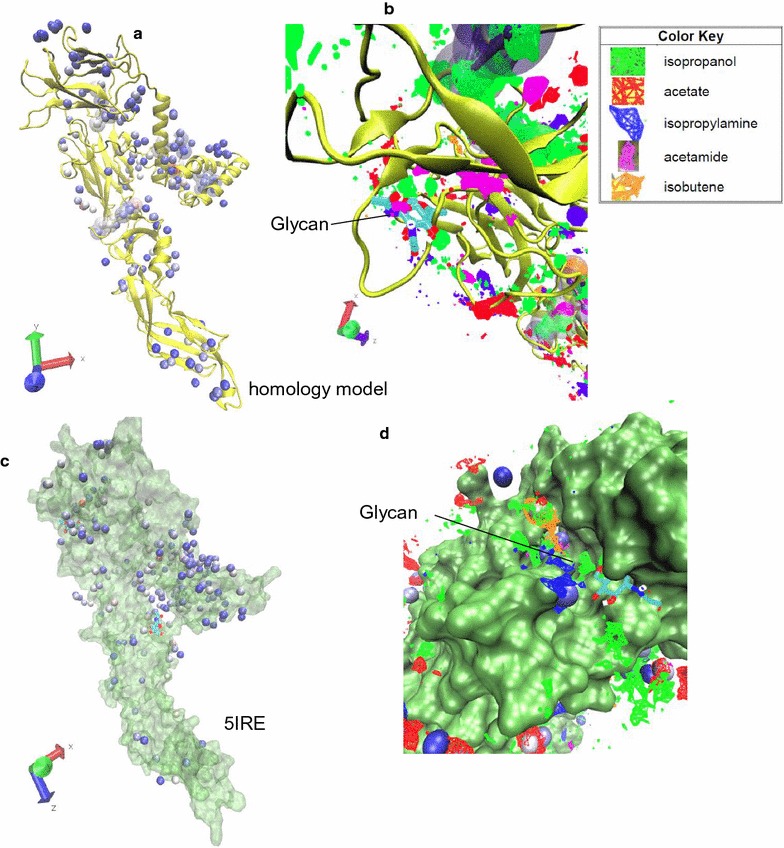
**a** The hotspots distribution across the ZVEP homology model; **b** the occupancy grid distribution of the probes at the receptor site suggesting the composition at each locale; **c** the hotspots distribution across the ZVEP 5IRE crystal structure; **d** the occupancy grid distribution of the probes at the 5IRE receptor site

Once the ligandable sites were revealed, druggability [21] of those sites was resolved by running NAMD [22] simulations of the protein receptor in the presence of small organic molecular probes. The probes, which are the most commonly used probes for druggability analyses [21] consisted of 60% isopropanol and equal 10% concentrations of isobutene, acetamide, acetate, and isopropylamine. The druggability assessment was performed with the intention of unraveling any “hot spots” or clusters of hotspots that indicate the existence of druggable receptors. The druggability analysis of the homology model revealed 162 small-molecule binding hotspots ranging from a minimum ∆G of −2.27 kcal/mol and maximum of −1.00 kcal/mol. The protein surface was enriched with 77 binding hotspots of isopropanol with the lowest binding free energy of −1.84 kcal/mol. Nevertheless, isobutene (33 hotspots, −1.90 kcal/mol), isopropylamine (17 hotspots; −1.98 kcal/mol), acetamide (11 hotspots, −2.27 kcal/mol), and acetate (24 hotspots, −2.00 kcal/mol) enrichment were more isolated. The analysis predicted the presence of the five druggable domains (Fig. 3a). The probe occupancy grid distribution across the ZVEP surface of the homology model at the vicinity of the receptor—suggesting active site compositions of a potential drug candidate—are depicted in Fig. 3b.

The hotspot distribution when the druggability analysis was applied to ZVEP 5IRE is depicted in Fig. 3c. The druggability analysis revealed 203 small-molecule binding hotspots ranging from a minimum ∆G of −2.34 kcal/mol and maximum of −1.00 kcal/mol. Throughout the protein surface, 105 binding hotspots of isopropanol was detected with the lowest binding free energy of −2.34 kcal/mol. Again, isobutene (51 hotspots, −1.85 kcal/mol), isopropylamine (15 hotspots; −1.94 kcal/mol), acetamide (6 hotspots, −1.90 kcal/mol), and acetate (26 hotspots, −1.99 kcal/mol) enrichment were more isolated. The analysis predicted the presence of two druggable domains (Fig. 3c). The probe occupancy grid distribution across the ZVEP surface of the homology model at the vicinity of the receptor—suggesting active site compositions of a potential drug candidate—are depicted in Fig. 3d.

The analysis suggests that the homology model predicts the potential druggability of ZVEP which was further confirmed by 5IRE. The primary active probe for ZVEP is isopropanol while the secondary being isobutene.

### Pharmacophore analysis

**Fig. 4:**
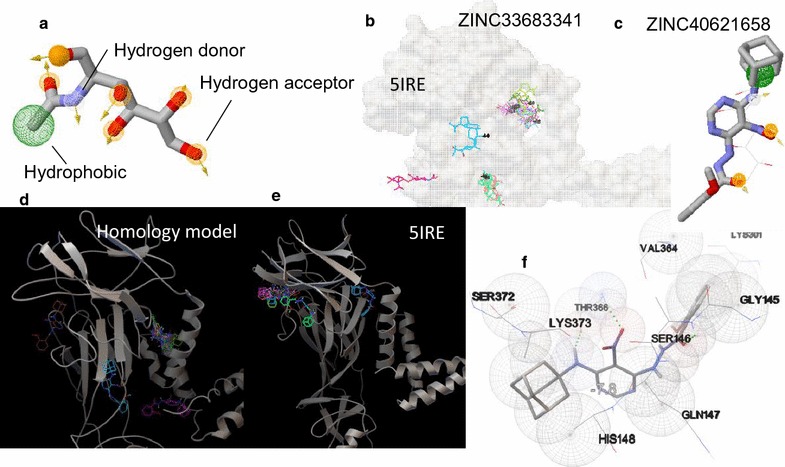
**a** Pharmacophore distribution of NAG; **b** ZINC 33683341 a potential inhibitor that was previously identified via homology model and efficacy verified via experimentation; **c** a potentially more potent viral inhibitor ligand screened using ELIXIR-A and Zincpharmar; **d** potential biding site distribution of ZINC 40621658 on homology model; **e** verification of binding sites on 5IRE; **f** interaction diagram depicting hydrogen bonding and close atom interactions of ZINC 40621658 with 5IRE

Pharmacophore analysis was done via Enhanced Ligand Exploration and Interaction Recognition Algorithm (ELIXIR-A) that is under development in our laboratory. The algorithm consists of a computational aided routine that recognizes pharmacophore points of the pharmacophore, i.e., the ensemble of steric, electrostatic and hydrophobic properties which is essential for optimal supramolecular interactions with the ZVEP receptor to inhibit its biological effect. In the case of NAG, the possible pharmacophore points are depicted in Fig. 4a. Then potential ligands were screened via Zincpharmer using: a combination of location of the functional groups (e.g. proton donor/acceptor, hydrophobic groups, positive/negative ion, and exclusion spheres); stabilization of the most effective conformation; Lipinski’s rule of five that defines properties necessary for good permeation [23] (i.e., the molecule having less than five proton-donators, the molecular weight is less than 500 Da, log P smaller than 5, the molecule having <10 acceptors, and the molecule using biological transporters so that the ligand does not attach too strong); and having at least three minimum pharmacophoric points.

Based on above criteria, four potential drug-like ligands were generated for the homology model: ZINC65541163 (N-[(1S)-1-methyl-3-phenyl-propyl]-1-[(3S)-tetrahydrofuran-3-yl]methanesulfonamide); ZINC40621658 (N′-[6-(1-adamantylamino)-5-nitro-pyrimidin-4-yl]-2-methoxy-benzohydrazide and two more ligands that were previously identified by our laboratory that were tested for their efficacy for Zika virus inhibition experimentally, i.e., ZINC ZINC33683341 ((1S,3aR,5aR,5bS,7aS,9R,11aR,11bR,13aS,13bS)-9-(1-hydroxy-1-methyl-ethyl)-1-isopropenyl-3a,5a,5b,11a-) and ZINC49605556 ((1R,2S)-1-(3,4-dimethoxyphenyl)-2-[4-[(2S,3R,4S,5R)-5-[4-[(1S,2R)-2-(3,4-dimethoxyphenyl)-2-hydroxy-) [12]. The characteristics of each candidate are depicted in Table 1.

**Table 1:** Chemical characteristics of select compounds screened via ELIXIR-A

Ligand	Affinity (kcal/mol) on homology model	Affinity (kcal/mol) on 5IRE	Mass	logP
NAG (substrate)	−5.0	−4.4	221	–
ZINC65541163	−6.1	−6.1	297	2.61
ZINC33683341	−7.3	−6.3/−5.9	456	7.7
ZINC40621658	−7.4	−7.8	438	4.35
ZINC49605556	−8.2	−7.5	732	5.75

All the four ZINC candidates depicted in Table 1 had higher binding affinities for the site than the glycan NAG—indicating that the ligands would bind tighter to the location as opposed to the glycan. It should be noted that the efficacy of ZINC33683341, the more conservative candidate of the three that has the highest affinities on 5IRE has been already analyzed using in vitro assay as described previously and verified to inhibit Zika virus [16] successfully. A close inspection of the binding confirmations of ZINC33683341 on 5IRE suggests the inhibition action is allosteric, i.e., although the ligand bound transverse to the primary glycan binding site (Fig. 4b), the binding action was able to trigger inhibition. Excitingly, based on binding affinities with the homology model and in silico verification of interactions on 5IRE, ZINC40621658 seems to hold even more promise (Fig. 4c–e). The distribution of ZINC40621658 (Fig. 4c) on 5IRE (Fig. 4e) is consistent with that predicted by the homology model (Fig. 4d). A close examination of the interaction diagram reveals that ZINC40621658 would bind at proximity to the glycan binding site and the inhibition would be allosteric (since all conformations of NAG make hydrogen bonding and close interactions with residues 153–154 on 5IRE as depicted in Fig. 2f, g whereas ZINC40621658 form interactions with several residues between residues 145–148 and 364–373. Due to more attractive affinities, spatial distribution, and desirable pharmacophore properties, ZINC40621658 has the potential to be a strong antiviral candidate for Zika virus inhibition and has to be experimentally verified for its efficacy.

## Conclusions

How ligands and small molecule drug-like probes interact with ZVEP was analyzed using a homology model developed using West Nile envelope protein as the template. The small molecular probes based study using the homology model and subsequently verified using actual experimental structure, 5IRE, revealed that ZVEP is druggable. A pharmacophore analysis followed by screening showed at least four ligands that allosterically binds to the glycan binding domain constituted by residues VAL 153 and ASN 154 in 5IRE. Based on further selection criteria ZINC40621658 was identified to have high potential to be a strong antiviral candidate for Zika virus inhibition and has to be experimentally verified for its efficacy. We believe that identification these compounds that have a high affinity to the glycan receptor is a decent starting point for drug discovery targeting ZVEP.

## Limitations

Of the five potential inhibitors that were screened, only one was experimentally verified. To ascertain the efficacy, all five should be subjected to experimental verification. Although this work targeted inhibition of the envelope, primarily due to advantages of the drug not being required to penetrate the virus, inhibition via this route requires relatively high ligand concentrations (~100 µM).
